# Micro-scaled high-throughput digestion of plant tissue samples for multi-elemental analysis

**DOI:** 10.1186/1746-4811-5-12

**Published:** 2009-09-26

**Authors:** Thomas H Hansen, Kristian H Laursen, Daniel P Persson, Pai Pedas, Søren Husted, Jan K Schjoerring

**Affiliations:** 1 Plant and Soil Science Laboratory, Department of Agriculture and Ecology, Faculty of Life Sciences, University of Copenhagen, Thorvaldsensvej 40, DK-1871 Frederiksberg C, Denmark

## Abstract

**Background:**

Quantitative multi-elemental analysis by inductively coupled plasma (ICP) spectrometry depends on a complete digestion of solid samples. However, fast and thorough sample digestion is a challenging analytical task which constitutes a bottleneck in modern multi-elemental analysis. Additional obstacles may be that sample quantities are limited and elemental concentrations low. In such cases, digestion in small volumes with minimum dilution and contamination is required in order to obtain high accuracy data.

**Results:**

We have developed a micro-scaled microwave digestion procedure and optimized it for accurate elemental profiling of plant materials (1-20 mg dry weight). A commercially available 64-position rotor with 5 ml disposable glass vials, originally designed for microwave-based parallel organic synthesis, was used as a platform for the digestion. The novel micro-scaled method was successfully validated by the use of various certified reference materials (CRM) with matrices rich in starch, lipid or protein. When the micro-scaled digestion procedure was applied on single rice grains or small batches of Arabidopsis seeds (1 mg, corresponding to approximately 50 seeds), the obtained elemental profiles closely matched those obtained by conventional analysis using digestion in large volume vessels. Accumulated elemental contents derived from separate analyses of rice grain fractions (aleurone, embryo and endosperm) closely matched the total content obtained by analysis of the whole rice grain.

**Conclusion:**

A high-throughput micro-scaled method has been developed which enables digestion of small quantities of plant samples for subsequent elemental profiling by ICP-spectrometry. The method constitutes a valuable tool for screening of mutants and transformants. In addition, the method facilitates studies of the distribution of essential trace elements between and within plant organs which is relevant for, *e.g*., breeding programmes aiming at improvement of the micronutrient density in edible plant parts. Compared to existing vial-in-vial systems, the new method developed here represents a significant methodological advancement in terms of higher capacity, reduced labour consumption, lower material costs, less contamination and, as a consequence, improved analytical accuracy following micro-scaled digestion of plant samples.

## Background

The concentration of essential plant nutrients must be maintained within certain concentration ranges in plant tissues. Analysis of the elemental composition is accordingly an important tool for diagnosis of nutritional disorders. Even before nutritional imbalances arise, changes in the elemental composition may constitute an important physiological indicator of plant responses to environmental parameters [1]. Also with respect to human nutrition, the content of essential trace elements, *e.g*. Fe and Zn, in vegetable food products is an important trait and large resources are devoted to improvement of the content and bio-availability of essential micro-nutrients in crops by bio-fortification [2-4].

Development of new genotypes of crop plants with improved stress tolerance and nutritional quality requires information about the genes and molecular processes underlying elemental transport and homeostasis in plants. Mutants and transformants of the plant model species Arabidopsis constitute a valuable tool for this purpose [5]. However, often a large number of lines have to be screened and a limited number of seeds may be available for elemental analysis. The same problem may occur in breeding programmes aiming at attracting new cereal lines with improved mineral composition by genetic modification or conventional breeding. In these cases, not only the total grain concentrations are relevant, but also the distribution of elements between grain tissues as well as their binding forms (speciation) [6]. Procedures for accurate analysis of the mineral composition in sub-grain tissue fractions available in limited quantity are therefore required.

Quantitative elemental analysis by ICP-spectrometry requires a complete digestion of solid samples in order to degrade the matrix components and obtain a homogenous liquid phase. The digestion of samples is usually the most time consuming step, thus constituting a bottleneck for high-throughput analysis and screening of large sample sets. Furthermore, when the amount of sample is limited and/or the elements of interest are present in trace or ultra-trace concentration, one of the main analytical challenges is to perform a rapid and thorough digestion in small volumes with minimum dilution and contamination [7-9].

Plant samples consist of an organic matrix that may cause analytical bias and block the sample introduction system if not fully decomposed. The most commonly used methods for destruction of organic matter are based on high-temperature-oxidation (dry ashing) or wet digestion in vessels using strong acids with or without the addition of oxidizing solvents. Wet digestion is usually carried out in a pressurised system using closed vessels heated in a microwave oven or autoclave [10-14]. Systems using closed vessels have several advantages relative to open systems, including significantly reduced risks of contamination and loss of volatile analytes [14,15]. In addition, the boiling point of the acid is elevated when the pressure inside the closed vessel increases, leading to a much faster and more thorough matrix digestion. Using a mixture of HNO_3 _and H_2_O_2_, most biological samples can be completely digested in closed vessels except if they have a high content of silicates. In such case, hydrogen fluoride must be added [16-18]. It is important to note that microwave HNO_3_/H_2_O_2 _digestion may not always provide a full recovery of elements such as Fe, Al and Se. Consequently, certified reference materials with a matrix comparable to the sample should always be included in the sample sets analysed.

The typical vessel volumes (50-120 ml) used in most conventional microwave rotors are too large for optimal digestion of small sample quantities [19]. In addition, the PTFE (polytetrafluoroethylene, TEFLON™) vessels used in these rotors require time consuming steps associated with liquid transfer to auto sampler vials. The PTFE vessels are expensive and need to be carefully cleaned between analytical cycles in order to avoid memory effects from the previous sample. A modified open vessel method using smaller polystyrene liners in an 80-position rotor allowed high-throughput digestion of small sample weights at low temperature [19,20]. For micro-scaling, various vial-in-vial setups have been employed to overcome the problem of incomplete digestion at low temperature [8,21-23]. However, physical limitation due to thick vial walls prevents high capacity rotors (48 positions) to be used. In addition, the PTFE screw thread frequently causes leakage problems.

Several analytical methods can be applied for elemental quantification of biological samples, including inductively coupled plasma-mass spectrometry (ICP-MS) and ICP-optical emission spectrometry (ICP-OES) [24,25]. The multi-elemental capacity in combination with an excellent precision and sensitivity (10^-18 ^g l^-1^) makes ICP-MS an ideal choice for analysis of digested plant samples. The use of micro-nebulization (<100 μl min^-1^) decreases the sample consumption so that the required volume of sample is reduced and simultaneous analysis of many elements made possible [26,27]. ICP-OES is an attractive alternative to ICP-MS as purchase prize and running costs are significantly lower. Moreover, the matrix tolerance and long-term stability of ICP-OES are usually better than that for ICP-MS, but at the expense of an inadequate sensitivity for several important trace elements occurring in plants, including the heavy metals Cd, Pb and Hg, and the essential plant micronutrients Ni and Mo.

The objective of the present work was to develop a high-throughput digestion method for multi-elemental analysis of plant samples available in small quantities. A commercially available 64-position rotor accommodating disposable glass vials, originally designed for microwave-based parallel organic synthesis [28] was used as platform for the digestion. The disposable glass vials were closed with special PFTE seals in polyetheretherketone (PEEK) screw caps allowing temperatures up to 200°C and pressures up to 20 bar, thus matching digestion conditions required for even fairly recalcitrant plant tissues such as seeds. The 64-position rotor also accommodated vials with dimensions fitting standard ICP auto samplers, thereby enabling direct measurement in the digestion vials without time consuming liquid transfer between containers. The use of small glass vials instead of a vial-in-vial system allowed fast heating in the microwave oven and subsequent cooling. By facilitating simultaneous digestion of many samples, the method developed here substantially decreases labour consumption.

The micro-scaled digestion method was validated using certified reference materials of durum wheat flour, apple leaves and bovine muscle, representing matrices differing in starch, lipids and protein contents. An accuracy better than 90% of the true value was achieved for the essential plant nutrients Cu, Fe, K, Mg, Mo, Mn, P, S and Zn, as well as for the elements Cd, Se and Al. Further validation of the developed micro-scaled digestion method was obtained by analysis of single rice seeds and their sub-fractions (endosperm, embryo and aleurone). Finally, it was shown that the elemental composition of small batches of Arabidopsis seeds (1 mg, corresponding to approx. 50 seeds) could be accurately determined.

## Results

### Analysis of certified reference materials

The efficiency of the developed micro-digestion procedure was evaluated for three very different certified reference materials, *viz*. apple leaf (CRM 1515), durum wheat flour (CRM 8436), and bovine muscle (CRM 8414). Beside differences in matrix composition, the three reference materials varied in elemental composition, spanning several decades of concentrations.

In apple leaves, very good agreement with the certified values was obtained for K, Ca, Mg, P, Al, Mn, Zn and Cu down to the lowest sample quantity of 1 mg (Table 1). Fe was in all cases 10-20% lower than the certified value. Similar underestimation was observed in a conventional large scale digestion (data not shown), suggesting a systematic bias not related to the micro-digestion procedure. The concentration of the trace element Mo agreed with the certified value for sample quantities of 5 mg, but not for smaller quantities. A similar observation was made for S, where a considerable over-estimation occurred below 5 mg sample weights.

**Table 1:** Accuracy of elemental concentrations in certified reference material of apple leaves (CRM 1515) measured after micro-scaled digestion.

**Element**	**Certified reference concentration μg g**^**-1**^	**Sample quantity, mg**					
		**1**	**2**	**5**	**10**	**15**	**20**
		**Accuracy (% of certified reference concentration)**					
K	16100 ± 200	91 ± 1	88 ± 1	95 ± 4	111 ± 2	115 ± 1	116 ± 1
Ca	15260 ± 150	100 ± 1	98 ± 2	97 ± 5	n.d.	n.d.	n.d.
Mg	2710 ± 80	100 ± 4	100 ± 5	96 ± 3	107 ± 2	110 ± 2	110 ± 1
S	1800 ± 192	244 ± 6	143 ± 2	111 ± 8	108 ± 2	109 ± 1	107 ± 2
P	1590 ± 220	99 ± 1	100 ± 3	98 ± 4	103 ± 3	104 ± 1	103 ± 2
Al	286 ± 9	100 ± 8	111 ± 12	95 ± 3	n.d.	n.d.	n.d.
Fe	83 ± 5	81 ± 1	80 ± 3	75 ± 4	92 ± 3	93 ± 2	90 ± 1
Mn	54 ± 3	100 ± 2	101 ± 1	100 ± 4	103 ± 2	106 ± 2	105 ± 1
Zn	12.5 ± 0.3	106 ± 3	95 ± 7	100 ± 5	104 ± 2	105 ± 1	103 ± 2
Cu	5.64 ± 0.24	90 ± 4	98 ± 1	99 ± 4	108 ± 3	107 ± 1	106 ± 1
Mo	0.094 ± 0.013	168 ± 12	132 ± 9	91 ± 4	n.d.	n.d.	n.d.

Values are means ± SE (n = 4).

Durum wheat flour is more difficult to digest than apple leaves due to a higher content of starch and lipids. This was illustrated by the fact that S was slightly underestimated even in the large scale digestion (Table 2). P, Mg, Mn, Zn, Se and Mo were measured accurately (less than ± 10% deviation from the certified value) down to sample quantities of 1 mg. In 2 mg samples, K, Fe and Cd were satisfactorily quantified for most sample weights. Particularly for Cd this was remarkable, considering the very low concentration of this element. As for the apple leaves, considerable over-estimation of S occurred in the analysis of 1 and 2 mg samples.

**Table 2:** Accuracy of elemental concentrations in certified reference material of durum wheat grain (CRM 8436) measured after micro-scaled digestion.

**Element**	**Certified reference concentration μg g**^**-1**^	**Sample quantity, mg**					
		**1**	**2**	**5**	**10**	**15**	**20**
		**Accuracy (% of certified reference concentration)**					
K	3180 ± 140	60 ± 1	91 ± 3	98 ± 2	93 ± 3	96 ± 2	96 ± 2
P	2900 ± 220	97 ± 2	84 ± 1	97 ± 2	90 ± 3	89 ± 1	89 ± 1
S	1930 ± 280	207 ± 4	123 ± 4	98 ± 2	86 ± 5	87 ± 2	87 ± 2
Mg	1070 ± 80	107 ± 4	97 ± 2	95 ± 1	95v2	99 ± 4	98 ± 1
Fe	41.5 ± 4	133 ± 20	97 ± 3	98 ± 7	92 ± 2	91 ± 2	91 ± 2
Mn	16 ± 1	96 ± 1	97 ± 1	97 ± 2	86 ± 2	93 ± 2	93 ± 2
Zn	12.5 ± 0.3	110 ± 2	99 ± 3	98 ± 1	97 ± 3	98 ± 3	98 ± 1
Cu	4.3 ± 0.69	45 ± 1	77 ± 5	91 ± 6	94 ± 6	97 ± 4	97 ± 4
Se	1.23 ± 0.09	100 ± 2	92 ± 4	93 ± 1	n.d.	n.d.	n.d.
Mo	0.7 ± 0.12	108 ± 13	98 ± 2	91 ± 2	102 ± 9	101 ± 7	92 ± 4
Cd	0.11 ± 0.05	137 ± 4	108 ± 2	95 ± 1	n.d.	n.d.	n.d.

Values are means ± SE (n = 4).

Samples of bovine muscle reference material were included as a representative of material with high protein content. The samples were analysed accurately for K, P, Na, Mg, Zn, Fe, Cu and Se even in the most extreme case where only 1 mg of sample was digested (Table 3). Also S was satisfactorily analysed, reflecting a higher concentration than in the reference materials consisting of apple leaf and durum wheat flour.

**Table 3:** Accuracy of elemental concentrations in certified reference material of bovine muscle (CRM 8414) measured after micro-scaled digestion.

**Element**	**Certified reference concentration μg g**^**-1**^	**Sample quantity, mg**					
		**1**	**2**	**5**	**10**	**15**	**20**
		**Accuracy (% of certified reference concentration)**					
K	15170 ± 370	95 ± 3	94 ± 3	95 ± 2	103 ± 1	102 ± 1	104 ± 1
P	8360 ± 450	101 ± 0.5	101 ± 0.6	101 ± 0.5	97 ± 1	95 ± 1	96 ± 2
S	7950 ± 410	121 ± 2	91 ± 0.4	91 ± 0.3	97 ± 1	96 ± 1	96 ± 2
Na	2100 ± 80	98 ± 6	91 ± 3	99 ± 3	119 ± 9	120 ± 8	112 ± 4
Mg	960 ± 95	99 ± 0.7	103 ± 0.6	100 ± 0.8	107 ± 1	107 ± 1	109 ± 2
Ca	145 ± 20	110 ± 9	110 ± 10	117 ± 2	n.d.	n.d.	n.d.
Zn	142 ± 14	98 ± 4	95 ± 4	100 ± 2	105 ± 1	104 ± 2	105 ± 3
Fe	71.2 ± 9.2	99 ± 4	95 ± 10	98 ± 2	101 ± 2	100 ± 2	100 ± 3
Cu	2.84 ± 0.45	109 ± 3	91 ± 3	102 ± 3	95 ± 2	95 ± 0	98 ± 3
Mo	0.08 ± 0.06	175 ± 34	131 ± 14	105 ± 5	n.d.	n.d.	n.d.
Se	0.076 ± 0.01	108 ± 6	97 ± 4	102 ± 2	n.d.	n.d.	n.d.

Values are means ± SE (n = 4).

### Analysis of Arabidopsis seeds

Analysis of the elemental composition of seeds of Arabidopsis mutants may often be restricted by the number of seeds available. The micro-scaled digestion method was therefore applied to seed batches of 1 mg, corresponding to approximately 50 seeds. The reproducibility of the results was generally very good, both for macro- and micro-nutrients, as shown by the low coefficient of variance of the mean values of four replicate batches (Table 4). Furthermore, it was possible to determine the concentration of Cd with only 3% variation following micro-scaled digestion despite the very low concentration of this element.

**Table 4:** Concentration of elements in seeds of wild type Arabidopsis (Col 0) analysed in batches of 1 mg (~50 seeds) or 20 mg (~1000 seeds).

**Element**	**Seed quantity analysed**			
	**1 mg**		**20 mg**	
	
	**Concentration** **μg g**^**-1**^	**%**	**Concentration** **μg g**^**-1**^	**%**

Mg	2301 ± 23	1	2638 ± 33	1
P	5987 ± 64	1	7258 ± 136	2
S	8799 ± 478	5	6084 ± 135	2
K	9817 ± 59	1	10946 ± 288	3
Ca	5069 ± 83	2	5736 ± 112	2
Mn	37 ± 1	3	43 ± 0.7	2
Fe	84 ± 3.7	4	75 ± 2	3
Cu	5.3 ± 0.2	3	5.6 ± 0.1	2
Zn	39 ± 1	3	47 ± 0.9	2
Mo	2.0 ± 0.02	1	2.0 ± 0.06	3
Cd	0.1 ± 0.004	3	0.1 ± 0.002	2

Values are means ± SE (n = 4).  denotes the coefficient of variance of the mean value.

**Figure 1:**
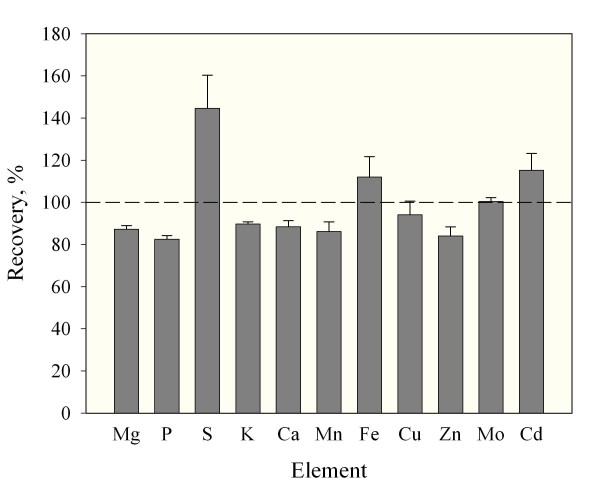
**Accuracy of elemental concentrations measured by ICP-MS on 1 mg micro-scale digested Arabidopsis seeds (~50 seeds) compared to the corresponding concentrations in a larger seed batch of 20 mg (~1000 seeds)**. Values are means + SE (n = 4).

The accuracy of the micro-scaled digestion method was evaluated based on comparison with data obtained from analysis of a larger seed batch (20 mg, corresponding to ~1000 seeds). Following micro-scaled digestion of 1 mg, there was a tendency to underestimate the concentration of several elements, but the difference was always <15% (Fig. 1). The largest deviation occurred for S which was overestimated by 40% following the micro-scaled digestion procedure (Fig. 1). This overestimation was of the same magnitude as in the analyses of the certified reference materials (see Table 3).

### Micro-scaled analysis of single rice grains

**Figure 2:**
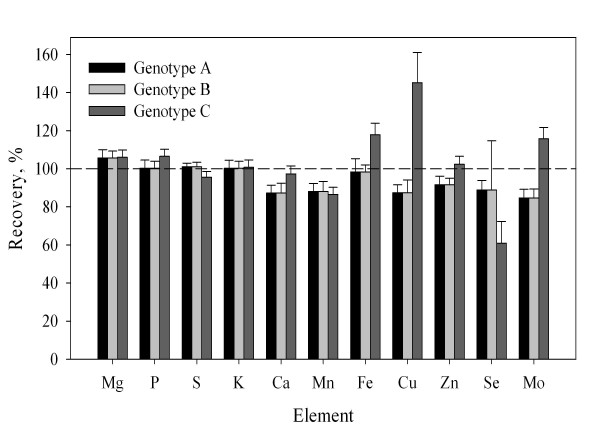
**Tissue concentrations of mineral elements in single grains of *Oryza sativa *L. cvs TSN1 (genotype A), Hom Nang Nouan (genotype B) and Kai Noi Leuang (genotype C)**. Values are expressed relative to data from macro-scaled analysis (250 mg) of finely pulverized grains of the same cultivars. Values are means + SE (n = 10).

Single, unpolished rice grains of three genotypes, on average weighing 21.6 ± 0.3 mg, were micro-scale digested without prior milling and analysed for their composition of essential macro- and micro-nutrients. The single grain analysis of Mg, P, S and K was accurate when compared to the corresponding results from macro-scaled analysis of finely pulverized grains of the same cultivars (Fig. 2). The Ca concentrations were slightly (~10%) underestimated. The same was the case for the micro-nutrients Mn and Mo, while Fe and Zn were more accurately determined (Fig. 2). In genotype C, Cu was significantly overestimated, while Se was underestimated. Part of the recorded variation may derive from sampling heterogeneities, indicating that 10 replicate seeds were not representative for a pulverized large batch. Taken as a whole, the micro-scaled digestion of single seeds were able to provide reliable result for most essential macro- and micro-nutrients in single rice grains.

### Micro-scaled analysis of rice grain tissues

Rice grains of the same three cultivars as used for the single grain analysis were fractionated into (*i*) an outer layer (aleurone incl. testa), constituting about 13% of total seed weight, (*ii*) the endosperm (~84% of seed weight) and (*iii*) the embryo, accounting for only ~2% of the seed weight. The individual fractions were subsequently analysed for Zn, Fe, P and S, which are all essential elements in human nutrition. The first two of these elements are particularly important due to their low bioavailability, causing human malnutrition on a global scale. The latter two elements are major constituents of phytic acid and proteins, respectively, potentially involved in Fe and Zn binding in the rice grain.

**Figure 3:**
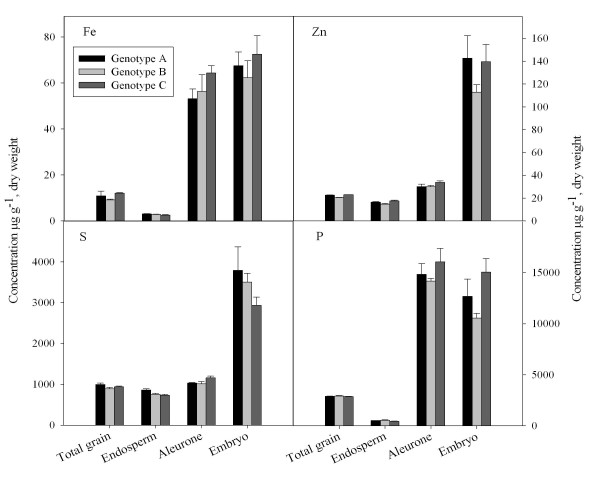
**Tissue concentrations of Zn, Fe, P and S in grain tissues of rice genotype A, B and C, Four rice grains per batch were separated into an outer aleurone layer (incl. testa), the endosperm and the embryo**. The three fractions were weighed, micro-scale digested and analysed by ICP-MS to obtain tissue elemental concentrations. Values are means + SE (n = 4).

The endosperm had a much lower concentration of the four analysed elements than the two other grain tissue fractions (Fig. 3). The highest concentrations of Zn were found in the embryo, particularly of genotypes A and C (Fig. 3). The aleurone layer had a Zn concentration which was approximately 2-fold higher than in the endosperm, but 4-fold lower than in the embryo. The embryo also contained the highest concentrations of S. The three genotypes had very similar Fe concentrations in the endosperm. Compared to Zn, Fe concentrations were considerably lower in all tissue fractions, including the endosperm (Fig. 3). The Fe, as well as the P concentration, was similar in the embryo and in the aleurone layer.

**Figure 4:**
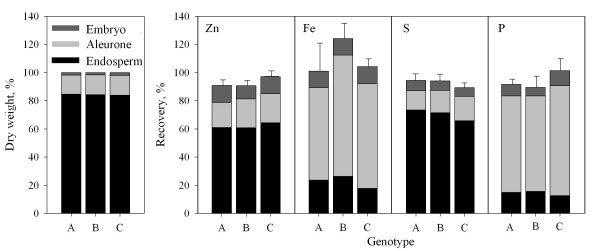
**Distribution of dry matter and Zn, Fe, P and S in grains of rice genotype A, B and C**. Rice grain was separated into an outer layer of aleurone (incl. testa), the endosperm and the embryo. The three fractions were weighed, micro-scale digested and analysed by ICP-MS to obtain tissue elemental concentrations. Elemental recoveries represent the summed up quantity in the three grain fractions relative to the total grain content derived from analysis of a large batch of pulverized grain material. Values are means + SE (n = 4).

The elemental recoveries following micro-scaled digestion of individual rice grain tissues were checked by a mass balance analysis in which the Zn, Fe, P and S concentrations in each of the three grain fractions were multiplied with the corresponding dry weights in order to estimate the quantity of each element in the individual tissue types. Thereafter, the resulting quantity for the three grain fractions was summed up and the cumulated recoveries compared with the total grain contents derived from analysis of a large batch of pulverized grain material. More than 90% of the Zn, Fe, P and S contained in the rice grain were recovered after dissection and micro-digestion of the individual tissue fractions (Fig. 4). Thus, the micro-scaled digestion procedure was able to provide reliable results for the distribution of the four investigated elements in a small quantity of rice grain. Zn and S were similarly distributed in the rice grain, with the endosperm being the most important compartment containing about 60-70% of the total content (Fig. 4). Conversely, the major part of Fe and P (>65%) was localized in the aleurone layer despite the fact that this compartment only constituted around 13% of the total grain weight. Only about 15-20% of the Fe was present in the endosperm. The embryo contained a similar proportion of all four elements (5-10%).

## Discussion

Three very different certified reference materials; *viz*. apple leaves, durum wheat flour and bovine muscle were used to evaluate the efficiency of the developed digestion procedure. These matrices differed considerably in digestibility, the apple leaves being most easily destructed due to their lower content of in particular starch, lipid and protein [29]. The three certified reference materials differed not only in their matrix composition but also in their physical texture with particle sizes ranging from 75 μm for apple leaf samples to >200 μm and >250 μm for durum wheat grains and bovine muscle, respectively. In micro-scaled digestion procedures, sample homogeneity is a very important factor, usually having a strong influence on the final results [21,30]. Some of the variation in the accuracies of the data presented in Tables 2 and 3 may thus derive from sampling since the particle unit size of the wheat grain material was relatively large (200 μm).

Even though the digestion procedure may result in a solution that appears fully transparent, residual carbon may still persist and cause analytical bias during the subsequent ICP analysis. One of the main reasons for this bias is that non-oxidized carbon acts as a surfactant, changing the efficiency of the nebulizer [31]. Moreover, a fluctuating carbon load to the plasma may interfere with the ionisation efficiency of analyte ions and create polyatomic interferences [32,33]. A minimum temperature of 300°C is needed for complete oxidation with nitric acid, while lower temperatures between 220 and 250°C result in residual carbon, although in low concentrations [34]. The addition of hydrogen peroxide to the digestion solution can lower the temperature requirements for total digestion. Nevertheless, most matrices still have to be digested in a closed vessel system in order to elevate the temperature above the boiling point [29]. In the present work, a 2:1 mixture of 70% nitric acid and 30% hydrogen peroxide made it possible to digest pulverized samples of bovine muscle and apple leaves at a temperature of 140°C. This was evidenced by the fact that the elemental analyses following digestion of 10 and 20 mg samples gave results matching the certified values (Tables 1 and 3). For durum wheat flour, a slight (11-14%) underestimation occurred for P and S, indicating incomplete digestion (Table 2). Reinforcing the lip seals with an extra seal and increasing the temperature above 140°C improved the recovery of P and S (data not shown), but also caused several vials to burst. Thus, vials with higher pressure resistance would be an advantage, but were not commercially available to fit the system. It has previously been shown that a temperature of 220°C during digestion of coal in vessels resulted in a pressure of 20 bar, which decreased to 9 bar upon lowering of the temperature to 180°C [20]. The standard glass vials used in our work had according to the manufacturer a pressure resistance of 20 bar (290 psi) but could still not resist temperatures >140°C. Thus, the matrix of the biological samples clearly behaved very differently from coal.

When small sample quantities are digested special care has to be taken in order to avoid contamination. The dilution factor for the 1 mg samples was more than 10 times higher compared to the large scale digestions and therefore the influence of contamination would have a greater influence on the results. For each individual element included in the analyses, the level of quantification (LOQ), *i.e*. the lowest concentration of a given target element that could be quantified on the ICP-MS, was calculated as 10 times the standard deviation of 7 blank samples. The LOQ values for *e.g*. S, P, Fe and Zn were 217, 20, 10 and 2.3 μg l^-1^, respectively. Following digestion of 1 mg samples, the resulting S concentration in the solution was 419, 391 and 1725 μg l^-1 ^for apple leaves, durum wheat grain and bovine muscle, respectively. The problems with over-estimation of S in the first two of these reference materials (Tables 1 and 2) were thus not exclusively caused by too low concentrations in the digest solution as these were higher than the LOQ.

Problems with overestimation of the S concentration also appeared in the micro-scaled digestion of 1 mg Arabidopsis seeds (Fig. 1), while for several other elements a tendency of slight underestimation (Fig. 1) was observed. This underestimation may represent sampling uncertainties.

All samples ≤ 5 mg were analysed by ICP-MS, while samples ≥ 10 mg were analysed by ICP-OES. The ICP-MS was equipped with a low consumption PFA nebulizer which minimises sample uptake to 0.1 ml min^-1 ^enabling more elements to be quantified when only a limited amount of sample is available. The ICP-OES was equipped with a Meinhard nebulizer with a sample flow of 1.5 ml min^-1^. The final diluted sample volume used for both ICP-MS and ICP-OES was 4.6 ml, enabling direct sample aspiration from the digestion vessels as these fitted into the standard auto samplers.

In addition to the analyses of certified reference materials, the micro-scaled digestion method was validated by a mass-balance approach in which the sum of the elemental contents determined for separate rice grain tissue fractions (aleurone, embryo and endosperm) was shown to satisfactorily match that obtained by analysis of whole rice grains (Fig. 3). The good overall recoveries obtained show that the micro-scaled method was able to digest samples between 1 and 20 mg accurately. In addition, the method was capable of dealing with different matrices, the embryo having a relatively high fat and protein content, the aleurone a relatively high protein content, and the endosperm a high starch concentration. The possibility of elemental profiling of sub-grain tissue fractions in small quantities will be a very valuable tool in future studies investigating how essential trace elements are distributed within the cereal grain. Such information is essential in relation to biotechnological strategies aiming at increasing, *e.g*., the Fe and Zn content in the endosperm [35]. The three different genotypes analysed in the present work, including two traditional Laotian varieties and a high yielding variety, did not exhibit remarkable differences, neither in total Fe and Zn concentrations, nor with respect to the distribution of these elements within the grain (Fig. 4). However, genotypic differences exist and breeding programmes attempting to select cultivars with elevated content of micro-nutrients are ongoing. For rapid screening of the elemental composition of grain tissues in such breeding programmes, the method developed and presented here will indeed be useful.

The micro-scaled digestion method made it possible to analyse single rice grain as evidenced by the good match between single seed concentrations and those obtained by analysis of a large batch of milled rice grain (Fig. 2). The elemental profiling of single rice grain showed that there was little variation in the macro-nutrient concentrations (Mg, P, S, K, Ca) between seeds in the same batch (Fig. 2). For micro-nutrients (Mn, Cu, Zn, Se and Mo) larger variability was recorded (Fig. 2). The possibility of analysing single grains of cereal species provides a valuable tool for high-throughput screening of breeding lines and transgenic rice lines. In addition, the micro-scaled digestion procedure will make it possible to follow the grain loading of essential minerals inside a single rice panicle, thereby providing information on the physiological controls and responses to changing environmental conditions.

## Conclusion

A micro-scaled method was developed which enables digestion of small quantities of plant samples for subsequent elemental profiling by ICP spectrometry. The method is based on a commercially available 64-position microwave rotor accommodating closed glass vials which are capable of withstanding sufficiently high temperatures and pressures to ensure digestion of even fairly recalcitrant plant tissues. The advantages of the new method compared to existing vial-in-vial methods are:

• The glass vials fit commercially available multi-position microwave rotors and standard ICP auto samplers, thereby enabling direct measurements without time consuming liquid transfer between vessels and without the accompanying risks of contamination.

• The glass vials allow fast and direct microwave heating and temperature control by IR sensors present in state-of-the-art microwave ovens, ensuring correct temperature readings and uniform thermodynamic conditions during the digestion. In contrast, the vial-in-vial procedure uses thermal convection, very often with water as ballast between the microwave bomb/liner and the vial to increase the thermal conductivity. This increases the heat capacity significantly and prolongs the time needed to heat the digestion units.

• Due to the low heat capacity of the vials they can be rapidly cooled which prevents loss of volatile elements before uncapping and facilitates a faster handling time.

• The glass vials are closed with polytetrafluoroethylene (PTFE) lip-seals in polyetheretherketone (PEEK) screw caps ensuring a thermo stabile and sealed digestion unit at elevated temperatures. In contrast, the PTFE screw threads used in most vial-in-vial systems are less stable.

• The design of the lip seals prevents explosions because they are only able to withstand a certain pressure.

• The current procedure allows a more cost- and labour-effective sample digestion because the glass vials are cheap and disposable. This eliminates time-consuming acid-based cleaning procedures between analytical runs and allows many samples to be prepared in advance.

The new method constitutes a valuable tool for high-throughput screening of e.g. mutants, transformants and breeding lines. In addition, the method facilitates studies of the distribution of essential trace elements between and within plant tissue fractions, which is highly relevant in relation to breeding programmes aiming at improved micro-nutrient density in edible plant parts.

## Methods

### Reference materials and plant samples

The following certified reference materials (CRMs) were used: NIST 8436 (durum wheat flour, particle size <200 μm), NIST 1515 (apple leaves, particle size <75 μm) and NIST 8414 (bovine muscle, particle size <250 μm). All of these were purchased from US Department of Commerce, National Institute of Standards and Technology, Gaithersburgh, MD, USA.

Brown rice grain (*Oryza sativa *L. cvs TSN1, Hom Nang Nouan and Kai Noi Leung) was supplied by the International Rice Research Institute (IRRI) as part of the Lao-IRRI Rice Research and Training Project and the EU-FP6 project META-PHOR . The two last genotypes represent traditional Laotian varieties and the first a high yielding variety. Seeds of Arabidopsis (*Arabidopsis thaliana*) were wild type (Col 0). Before analysis, rice grains and Arabidopsis seeds were rapidly rinsed 3 times in Milli-Q water (18.2 Ω; Milli-Q Plus, Millipore Corporation, MA. USA) and subsequently freeze dried at 0.5 mbar for 24 h (Christ Alpha 2-4, Martin Christ GmbH, Osterode, Germany).

The rice grains were sectioned into three main tissue fractions: (1) The bran layers (including pericarp, testa and aleurone), (2) the embryo (including the scutellum) and (3) the endosperm. First, the embryo was gently loosened and removed by use of the tip of a scalpel. To separate the bran and the endosperm, a polishing process was performed by high speed shaking in a ball mill (Retsch MM301). The mill was operated at 30 Hz for 120 s and mounted with a rack containing micro-centrifuge tubes with 200 mg of acid washed quartz sand and 4 rice grains. The mixture of sand and abraded material from the grain was collected as bran layers. The remains of the grain (endosperm) were washed three times with milli-Q water to remove surface dust and dried.

### Micro-scaled digestion

**Figure 5:**
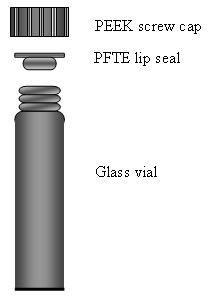
**Disposable glass vials closed with polytetrafluoroethylene (PFTE) lip-seals in polyetheretherketone (PEEK) screw caps ensuring a thermo stabile and tight digestion bomb at elevated temperatures**.

Using a high-accuracy balance (Mettler Toledo MT5), between 1 and 20 mg of the three certified reference materials were weighed and transferred to digestion tubes consisting of disposable standard glass vials (Wheaton^® ^15 × 46 mm, Cap 13-425). A mixture of 125 μL 30% H_2_O_2 _(J.T. Baker, The Netherlands) and 250 μL 65% HNO_3 _(Sigma-Aldrich, UK) was added to each vial containing ≤ 5 mg material, while the double volume of reagents was used for the higher sample quantities. To ensure tightness and stability at elevated temperatures, the vials were closed with special PEEK screw caps (MG5, Anton Paar GmbH, Graz, Austria) and disposable PFTE lip-type seals (Mat. No. 41186, Anton Paar GmbH, Graz, Austria; Fig. 5). Sample digestion was carried out in a microwave oven (Multiwave 3000, mode: Synthos, Software version 2.01, Anton Paar GmbH, Graz, Austria) mounted with a 64 position carousel (64MG5, Anton Paar GmbH, Graz, Austria). A 10 min ramping period was used to reach a digestion temperature of 140°C, which thereupon was maintained for 80 min. After cooling for 10 min, the vials were put in a freezer before releasing the pressure by uncapping. The freezing step ensured better recovery of volatile elements such as S and Se. Finally, samples were diluted to a final concentration of 3.5 or 7% HNO_3 _(4.6 ml) and analysed directly in the vial.

Rice grain materials and Arabidopsis seeds were digested by the same procedure as used for certified reference materials except that the micro-wave digestion period was elongated with 30 min, now lasting 110 min instead of 80 min. Traditional large-scale digestion of 250 mg CRM and milled rice grain samples was carried out for comparison with the micro-scaled digestion procedure. Samples were micro-waved for 50 min at 210°C at a maximum pressure of 40 bar in 100 ml closed tubes. The digestion medium consisted of 5 ml 65% HNO_3 _and 5 ml 15% H_2_O_2_.

### Instrumentation for multi-elemental analysis

Multi-elemental analysis of samples weighing ≤ 5 mg was performed using ICP-MS (Agilent 7500ce, Agilent Technologies, UK) equipped with a PFA micro-flow nebulizer. ICP-MS chromatographic data were processed using Plasma Chromatographic Software v. B-03-07 (Agilent Technologies, UK). The ICP-MS was tuned in standard mode (no reaction/collision gas used) to achieve a sensitivity higher than 18000, 36000 and 18000 cps ppb^-1 ^on the masses ^7^Li, ^89^Y and ^205^Tl, respectively, while at the same time ensuring that the oxide level at m/z 156/140 was below 0.5%. The plasma power was operated at 1450 ± 50 W and the carrier and make-up gases were typically set at 0.83 and 0.17 l min^-1^. Sample uptake was maintained at approximately 0.1 ml min^-1 ^by the self-aspirating PFA nebulizer. Larger samples quantities (≥ 10 mg) were analysed by ICP-OES (Optima 5300 DV, PerkinElmer, USA) equipped with a Meinhard nebulizer and a cyclonic spray chamber. The RF Power was 1400 W and nebulizer and auxiliary flows 0.65 and 0.2 l min^-1^. Sample flow was set at 1.5 ml min^-1^. ICP-OES data was processed using Winlab 32 (Ver. 3.1.0.0107, PerkinElmer, USA).

## Competing interests

The authors declare that they have no competing interests.

## Authors' contributions

THH, KHL, DPP and PP carried out the experimental work. SHU and JKS conceived the study and devised the experimental design. THH, SHU and JKS wrote the manuscript. All authors read and approved the final manuscript.
